# Determinants of timely initiation of complementary feeding among mothers with children aged 6–23 months in Lalibela District, Northeast Ethiopia, 2015

**DOI:** 10.1186/s12889-016-3566-z

**Published:** 2016-08-25

**Authors:** Wondimu Sisay, Melkie Edris, Amare Tariku

**Affiliations:** 1 Department of Reproductive and Child Health, Institute of Public Health, College of Medicine and Health Sciences, University of Gondar, Gondar, Ethiopia; 2 Department of Human Nutrition, Institute of Public Health, College of Medicine and Health Sciences, University of Gondar, Gondar, Ethiopia

**Keywords:** Complementary feeding, Children aged 6–23 months, Determinants, Northeast Ethiopia

## Abstract

**Background:**

Optimal complementary feeding alone prevents six percent of child mortality, but it has continued to be considered as sub-optimal in Ethiopia. Therefore, this study aimed to assess timely initiation of complementary feeding and associated factors among mothers with children aged 6–23 months in Lalibela District.

**Methods:**

A community-based cross-sectional study was conducted from March 01 to April 29, 2015. Four hundred twenty-one mother-child pairs were selected by the systematic random sampling technique. An interviewer-administered questionnaire was used to collect data. A multivariable logistic regression analysis was employed to identify factors associated with timely initiation of complementary feeding. The Adjusted Odds Ratio (AOR) with a 95 % Confidence Interval (CI) was computed to assess the strength of association, and variables with a *P*-value of <0.05 were considered as statistically significant in the multivariable analysis.

**Results:**

The study demonstrated that, the prevalence of timely initiation of complementary feeding was 63 %. In addition, mother’s education [AOR = 4.33, 95 % CI: 1.99, 9.40], antenatal care follow up [AOR = 5.90, 95 % CI: 2.45, 14.21], and institutional delivery [AOR = 2.54, 95 % CI: 1.33, 4.82] were found key determinants of timely initiation of complementary feeding.

**Conclusion:**

In this community, timely initiation of complementary feeding was lower than the World Health Organization cut-off point for good practice of complementary feeding. Therefore, intensifying utilization of antenatal care and institutional delivery helps to improve the coverage of timely initiation of complementary feeding. Furthermore, the focus needs to be on uneducated women.

## Background

Around the age of 6 months, infant needs for energy and micronutrients start to exceed what is provided by breast milk. They are developmentally ready to initiate additional (complementary) food, which is necessary to meet their extra energy and micronutrient requirement [1]. In addition, the transition period (6 months to 2 years) is part of the ‘critical window of opportunity’ to enhance the survival and optimal growth of the child [2]. Thus, the World Health Organization (WHO) recommends that mothers should initiate soft, semi-solid, or solid food to their infants at the age of 6 months [3].

Infants and young children bear the heaviest burden of undernutrition and continue to suffer from disability and death associated with it [4]. Globally, undernutrition results in 3 million child deaths annually and this amounts to 45 % of all causes of mortality. Over two-thirds of these deaths are often associated with inappropriate feeding practice and occur during the first year of life [5, 6]. Sub-optimal breastfeeding results in more than 800,000 deaths annually [6] and is also a significant determinant of childhood undernutrition [7, 8]. On the other hand, optimal breastfeeding prevents 13 % of the deaths occurring in children under five, and appropriate complementary feeding results in an additional six percent reduction [9].

Nevertheless, global complementary feeding practice has been sub-optimal. Among South Asian countries, the rate of timely initiation of complementary feeding is lower than the WHO recommendation for good practice (80–94 %) [10]. In this regard, about 71 %, 70 %, 55 %, and 39 % of the infants in Bangladesh, Nepal, India, and Pakistan, respectively, are reported to have timely initiation of complementary feeding [8, 10–12]. On the other hand, there is a low rate of timely initiation of complementary feeding in Africa [1, 13]. In Ethiopia, more than half (57 %) of the child mortality occurs mainly due to undernutrition [14], and the majority of the children have had sub-optimal feeding practices. Only51% of the infants aged 6–9 months receive complementary food [15]. Furthermore, studies from different regions of the country show a low rate of timely initiation of complementary feeding (52.8–62.8 %) [16–18].

The determinants of timely initiation of complementary feeding vary between settings mainly depending on the level of health care utilization and socio-demographic characteristics. Reports from different countries reveal that, child sex, mothers wealth status, marital status, maternal and paternal education, maternal age (≥30 years), exposure to media, and knowledge about the right time for initiation of complementary feeding [11, 18–23], Antenatal Care (ANC) follow up, postnatal care, and institutional delivery [8, 11, 18, 20, 21, 23] are the commonly reported determinants of timely initiation of complementary feeding.

In order to reduce the high burden of child malnutrition and mortality in Ethiopia [15], ensuring appropriate Infant and Young Child Feeding (IYCF) practices is of vital importance. The country has implemented the IYCF strategy for a decade [24], so, studies showing complementary feeding practices have a paramount significance in evaluating the progress of interventions aiming to address inappropriate child feeding practices. However, such studies are scarce in Ethiopia, particularly in the northeastern part of the country. Therefore, this study aimed to assess the timely initiation of complementary feeding and its determinants among mothers with children aged 6–23 months in Lalibela District, northeast Ethiopia.

## Methods

### Study design and setting

A community-based cross-sectional study was conducted from March 01 to April 29, 2015, in Lalibela District, northeast Ethiopia. The district is one of the historical tourist destinations registered by the United Nations Educational, Scientific, and Cultural Organization (UNESCO) as a world wonder site. Lalibela District, which is 700 km from Addis Ababa, the capital city of Ethiopia, has two urban and one rural kebeles (smallest administration units in Ethiopia). According to the 2014/15 District Finance and Economic Development Office annual statistical report, the total population of the district was 31,491, of which children aged 6–23 months comprised 4.39 % (1065). The total number of households in the district was estimated at 7323. At the moment, one district hospital, one health center, two urban and one rural health posts were providing health services to the community.

### Sample size and sampling procedure

All mothers with children aged 6–23 months and have lived in the three kebeles of Lalibela District for at least 6 months were eligible for the study. The sample size was determined using single population proportion formula by considering the following assumptions: 52.8 % as a proportion of mothers practicing timely initiation of complementary feeding [18]; 5 % margin of error, and 95 % confidence level. In addition, a 10 % non-response rate which gives the final sample size of 421was anticipated. Information regarding the total number of households with eligible children was obtained from the District Health Office. The number of households sampled from the three kebeles of the district was proportionate-to-population size. A systematic random sampling technique was used to select individual households with eligible children. A sampling interval was calculated by dividing the total number of mothers who had children aged 6–23 months during the study period by the allocated sample size. For households with multiple children, one child was selected using the lottery method.

### Data collection tools and procedures

A structured interviewer-administered questionnaire was used to collect data. To maintain consistency, the questionnaire was first translated from English to Amharic (the native language of the study area) and was back-translated to English by professional translators. Six female clinical nurses and two health officers who were not working in the actual study area were recruited as data collectors and supervisors, respectively. Training was given to data collectors and supervisors for 2 days on basic skills of interview, ways of obtaining consent, and on how to maintain confidentiality of information. The investigators oversaw all data collection activities. The completion, accuracy, and clarity of the collected data were checked carefully on daily basis. A pre-test was administered on 5 % (21) of the sample out of the study area. During the pre-test, the acceptability and applicability of the procedures and tools were evaluated, but the result of the pre-test was not included for analysis.

Complementary feeding practices were assessed according to the key indicators recommended by WHO [3]. Accordingly, the outcome variable (timely initiation of complementary feeding) was confirmed by asking the mothers to recall the age at which they initiated additional food to [child’s name], and were asked “When did you first introduce any solid, semi-solid, or fluid to [child’s name] in addition to breast milk”. Then, if the mother answered “at the sixth month”, it was categorized as timely initiation of complementary feeding and coded as “1”, but if she had initiated beyond (before or after) the sixth month, it was considered as untimely initiation of complementary feeding and was coded as “0”. In addition, a single visit was made to gather data regarding complementary feeding practices and other characteristics. For a few mothers who faced difficulties in remembering the right time of initiation of complementary feeding, data collectors carried out different probing mechanisms to help them recall, thereby minimizing recall bias. Relating the time of initiation to known public events, occurrences of common childhood developmental milestones, and immunization schedules were some of the probing techniques used by data collectors. Moreover, to minimize the social desirability bias, data collectors created a conducive environment by keeping mothers apart and making them comfortable during data collection.

The standardized Dietary Diversity Score (DDS) tool with a 24 h recall was used to qualitatively assess the dietary intake of children. Mothers were asked to list the food items consumed by the children in the previous 24 h preceding the date of survey. The food items were categorized into seven food groups, such as starchy staples (grains, roots, and tubers), legumes and nuts, dairy products, flesh food (meat, fish, poultry, and organ meat), egg, vitamin-A rich fruits and vegetables, and other fruits and vegetables. By considering the minimum acceptable DDS, a child having a DDS of ≥4 was categorized as good dietary diversity, but if it had a DDS of <4, it was considered as poor dietary diversity [3].

The independent variables included in the study were mainly related to the parents’ socio-demographic and economic characteristics (age, education, employment, household wealth, religion, household size, and marital status), and health-care related information (ANC, place of delivery, postnatal care, frequency of health extension visits, and mothers’ knowledge and source of information about the right time for initiation of complementary feeding). Accordingly, to determine their knowledge about the right time for initiation of complementary feeding, the mothers were asked such a key question as “What is the appropriate time to start additional food for a child in addition to breastfeeding?”. If the mother said “at the sixth month”, she was considered as having accurate information about the right time for initiation of complementary feeding; otherwise, she was deemed to have inaccurate information. The Household Wealth Index was computed using a composite indicator by considering properties, such as livestock ownership, selected household assets, and size of agricultural land. Principal Component Analysis was performed to categorize household wealth status into poor, medium, and rich.

### Data analysis

The collected data were entered into the EPI-Info version 7 software, and transferred, cleaned, coded, and analyzed using Statistical package for Social Science (SPSS) version 20. Descriptive statistics, including frequencies, proportions, means, and standard deviations were used to summarize the variables. A binary logistic regression was used to identify the determinants of timely initiation of complementary feeding. A bivariable analysis was done to show the crude effect of each independent variable on the outcome variable. Variables with a *P*-value of <0.2 in the bivariable analysis were entered to a multivariable logistic regression analysis. Stepwise backward Likelihood Ratio (LR) was used for multivariable logistic regression. The Adjusted Odds Ratio (AOR) with a 95 % Confidence Interval (CI) was computed to assess the strength of association, and a *P*-value of <0.05 was used to declare the statistical significance in the multivariable analysis.

## Results

### Socio-demographic and economic characteristics

A total of 421 mother-child pairs were included in the study. The mean age (±Standard Deviation, SD) of the children was 14.87(±4.73) months. The majority of the respondents (95.2 %) were Orthodox Christians and 83.4 % of them were married. All of the participants were Amhara by ethnicity. More than half (52.3 %) of the children were male, and nearly one-third (27.8 %) were in the age range of 6 to 11 months. About 28.5 % of the mothers had no formal education, and more than half (59.9 %) were housewives (Table 1).

**Table 1:** Socio-demographic and economic characteristics of mothers with children aged 6-23 months in Lalibela District, northeast Ethiopia, 2015

Variables	Frequency	Percent
Child age in months		
6–11	117	27.8
12–17	150	35.6
18–23	154	36.6
Sex of child		
Female	201	47.7
Male	220	52.3
Relationship of care giver to the child		
Mother	413	98.1
Other ^a^	8	1.9
Maternal age		
15–24	120	28.5
25–34	216	51.3
> 34	85	20.2
Maternal religion		
Orthodox	401	95.2
Other ^b^	20	4.8
Maternal marital status		
Currently married	351	83.4
Currently unmarried ^c^	70	16.6
Maternal education		
Uneducated	120	28.5
Primary school	106	25.2
Secondary school and above	195	46.3
Maternal occupation		
Employed ^d^	70	16.6
Housewife	252	59.9
Others ^e^	99	23.5
Paternal education		
Uneducated	105	30.0
Primary school	51	14.5
Secondary school and above	195	55.5
Paternal occupation		
Employed	163	38.7
Merchant	58	13.8
Farmer	51	12.1
Unemployed	79	18.8
Family size		
2–3	155	36.8
4–6	245	58.2
> 6	21	5.0
Possession of microfinance bank account		
Yes	251	59.6
No	170	40.4
Wealth status		
Poor	139	33.0
Medium	189	44.9
Rich	93	22.1

^**a**^ Grandmother and sister

^**b**^ Muslim and protestant

^**c**^ Single, divorced, widowed

^d^ Governmental and non-governmental employees

^**e**^ Student, farmer, unemployed, merchant, daily laborer

### Maternal health care related characteristics

Nearly three-fourths (74.1 %) of the mothers had at least one ANC visit for the index child, of which about 81.7 % had 3 to 4 visits. Nearly two-thirds (63.4 %) of them gave birth at health institutions, and about one-quarter (25.4 %) had postnatal care (Table 2).

**Table 2:** Maternal health care and child feeding practices in Lalibela District, northeast Ethiopia, 2015

Variables	Frequency	Percent
Antenatal care (413)		
Yes	306	74.1
No	107	25.9
Frequency of antenatal care visits		
1–2 times	30	9.8
3–4 times	250	81.7
≥ 5 times	26	8.5
Place of delivery (413)		
Health facility	262	63.4
Home	151	36.6
Postnatal visit (413)		
Yes	105	25.4
No	308	74.6
Frequency of feeding per day (*n* = 411)		
1–2 times	94	22.3
3 times	190	45.1
≥ 4 times	127	30.2
Dietary diversity score (*n* = 411)		
Poor	360	87.6
Good	51	12.4
Type of food at first time of weaning		
Gruel	385	91.4
Porridge	364	86.5
Cow milk	112	26.6
Powder milk	50	11.9
Others ^a^	12	3.3
Information on TICF ^b^		
Accurate information	248	58.9
Inaccurate information	173	41.1
Source of information on TICF		
Health extension workers	350	83.1
Health care professionals	302	71.7
Television and radio	151	35.8
Community health worker	23	5.5

Since ten children didn’t initiate complementary feeding until the date of survey, the total number of children within variables for feeding frequency and dietary diversity is not 421

^a^ Tea, sugar water and juice

^b^ Time for initiation of complementary feeding

### Complementary feeding practices

The prevalence of timely initiation of complementary feeding was 63 % [95 % Cl: 58.0, 67.5 %]. One hundred eighteen children (28.0 %) started complementary feeding after sixth months, while twenty-eight (6.7 %) were initiated before they reached the sixth month. In addition, ten children (2.3 %) were offered no additional food since they have been born. Nearly two-thirds (58.9 %) of the mothers had accurate information about the right time of initiation of complementary feeding. The majority (87.6 %) of the children had poor dietary diversity, and below half (45.1 %) ate three times a day (Table 2).

### Factors associated with timely initiation of complementary feeding

In the bivariable analysis, maternal education, occupation, marital status, wealth status, paternal education and occupation, possession of television, ANC follow up, place of delivery, postnatal care, and health extension visits in the past 6 months were significantly associated with timely initiation of complementary feeding.

However, the result of the multivariable analysis showed that maternal educational status, ANC follow-up, and place of delivery were significantly and independently associated with timely initiation of complementary feeding. Accordingly, the likelihood of timely initiation of complementary feeding among children whose mothers attended primary school [AOR = 3.39, 95 % CI: 1.52, 7.54] and secondary school and above [AOR = 4.33, 95 % CI: 1.99, 9.40] was higher compared to that of uneducated mothers. Moreover, increased odds of timely initiation of complementary feeding were noted among mothers who had ANC follow up [AOR = 5.90, 95 % CI: 2.45, 14.21] compared to those had no ANC follow up. Similarly, the odds of timely initiation of complementary feeding were higher among mothers who gave birth at health facilities [AOR = 2.54, 95 % CI: 1.33, 4.82] as compared to those who gave birth at home (Table 3).

**Table 3:** Factors associated with timely initiation of complementary feeding among mothers with children aged 6–23 months in Lalibela District, northeast Ethiopia, 2015

Variables	Timely initiation of complementary feeding		COR ^c^(95 % CI)	AOR ^d^(95 % CI)
	Yes#	No#		
Maternal age				
25–34	140	76	0.92(0.58,1.48)	
> 34	45	40	0.56 (0.32,0.99)	
15–24	80	40	1	
Maternal marital status				
Currently married	228	123	1.65(0.99,2.78)	
Currently unmarried	37	33	1	
Maternal education				
Illiterate	41	79	1	
Primary school	63	43	2.82(1.64,4.85)	3.39(1.52,7.54) ^b^
Secondary school and above	161	34	9.12(5.38,15.48)	4.33 (1.99, 9.40) ^b^
Maternal occupation				
Employed	55	15	2.3 (1.25, 4.36)	
Others	56	43	0.83 (0.57, 1.32)	
Housewife	154	98	1	
Wealth status				
Poor	71	68	1	
Medium	145	44	3.16 (1.97,5.07)	
Rich	49	44	1.07 (0.63,1.80)	
Paternal education				
Illiterate	45	60	1	
Primary school	31	20	2.07(1.05, 4.09)	
Secondary school and above	152	43	4.71(2.82, 7.88)	
Paternal occupation				
Employed	121	42	2.55(1.59, 4.11)	
Merchant	40	18	1.97(1.04, 3.74)	
Farmer	25	26	0.85(0.45, 1.61)	
Unemployed	79	70	1	
Owning television				
Yes	141	45	2.81(1.84, 4.28)	
No	124	111	1	
Antenatal care				
Yes	245	61	21.26(11.80,38.33)	5.9(2.45,14.21) ^b^
No	17	90	1	1
Place of delivery				
Yes	206	56	6.24 (4.01,9.72)	2.54(1.33,4.82) ^b^
No	56	95	1	1
Postnatal care				
Yes	80	25	2.22(1.34,3.67)	
No	182	126	1	
Health extension visits ^a^				
No visit	10	21	1	
1–2 times	85	69	2.59 (1.14,5.86)	
3–4 times	147	60	5.15 (2.29,11.57)	
≥ 5 times	23	6	8.05 (2.49,25.99)	

^a^ health extension visits were determined based on the number of visits made during the past 6 months preceding the date of survey

^b^ significant at a *P*-vale of <0.05

^c^ Crude Odds Ratio

^d^ Adjusted Odds Ratio

## Discussion

The result of this study revealed that the prevalence of timely initiation of complementary feeding was 63 %. This finding was lower than the WHO cut-off point (80 % to 94 %) for good practice of complementary feeding [10]. Similarly, it was lower than the reports from Abyi-Adi, Ethiopia (79.7 %) [25], India (77.5 %) [26], and Bangladesh (71 %) [27]. The higher prevalence of timely initiation of complementary feeding in the latter study areas could be related to the improvements in utilization of ANC and institutional delivery. Hence, nutrition education and counseling are components of maternal health care services; a higher utilization of these services will bring an added benefit to improve mothers’ awareness on appropriate child feeding practices [25].

However, our finding was higher than that of the 2011 Ethiopian Demographic Health Survey report (51 %) [15] and the report from northern Ethiopia (52.8 %) [18]. This is probably related to the current improvements in the implementation of the Health Extension Program. Health extension workers are making home to home visits on regular bases to support families in accessing basic health services and to give home-based health education as well as other promotion services, including promotion of appropriate IYCF.

Compared to the current finding, a lower prevalence of timely initiation of complementary feeding was reported from Nigeria (41 %) [13] and India (55.7 %) [12]. This discrepancy could be attributed to differences in sample sizes between the two study areas, as more mothers were surveyed in India than in this study. Though the sample size was lower in Nigeria, a lower practice of timely initiation of complementary feeding could be related to the variation in mothers’ working environments; in Nigeria, most of the mothers were working outside the home, while most mothers were housewives in Ethiopia. It was affirmed that mothers working outside their home were more likely to return to work before they exclusively breastfed their infants for the recommended duration, 6 months [28–30]. As a result, they would be forced to initiate complementary feeding to their infants earlier than housewife mothers.

The result of the adjusted analysis showed that the odds of timely initiation of complementary feeding were higher among educated mothers as compared to their uneducated counterparts. Parallel findings were reported by studies elsewhere [16–18, 25, 31]. This could be due to the fact that education has an immense benefit in promoting the optimal maternal nutrition and health seeking behavior [15]. In addition, other studies persistently claimed that mothers’ education has a profound effect on children’s nutritional status and caring practice [15, 32–34].

Higher odds of timely initiation of complementary feeding were observed among mothers who visited ANC as compared to those who did not. This finding was in agreement with other reports in Ethiopia [16–18, 25]. Obviously, pregnancy has been considered as an important window of opportunity to deliver nutrition counseling and education on IYCF [35]. Nutrition education and counseling have a pivotal role in enhancing mothers’ IYCF knowledge and practice and maternal self-efficacy in child care [36–38].

Mothers who delivered at health institutions were more likely to initiate complementary feeding at the right time (sixth month) as compared to those who gave birth at home. The finding was supported by reports elsewhere [16–28]. Mothers who give birth at health institutions have a better opportunity to access appropriate child feeding information, which ultimately improves their capacity to challenge unfavorable attitudes of the community [35, 39]. The previous reports also claimed that institutional delivery was found to increase the likelihood of exclusive breastfeeding for 6 months, indicating timely initiation of complementary feeding [28, 40, 41]. On the other hand, home birthing is associated with inappropriate neonatal feeding practices, such as discarding colostrum and giving prelacteal feeds [42–44].

The investigators made a lot of effort to maintain the quality of the data, mainly through a pretest, frequent field supervisions, and training of data collectors. However, the study was not free from some limitations. First, since the estimation of some of the variables, including health extension visits, was made through recall (with the longest recall period of 6 months), there might have been a risk of recall bias. Secondly, the study did not consider the measurement of some of the independent variables, like type of nutrition services received during ANC and postnatal care visits, and mothers’ attitude towards appropriate breastfeeding and complementary feeding which might confound the result of the study. Thirdly, the study was not free from the pitfalls of a cross-sectional study design.

## Conclusion

This study revealed that, the prevalence of timely initiation of complementary feeding was low; it was by far lower than the WHO cut-off point for good practice of complementary feeding. Mother’s educational status, ANC follow up, and institutional delivery were significantly associated with timely initiation of complementary feeding. Therefore, current efforts should be strengthened to improve optimal complementary feeding practices by stepping-up the utilization of ANC and institutional delivery. The focus needs to be on uneducated women. Furthermore, future studies should emphasize mixed methods, such as triangulating with a qualitative study design and prospective study designs to identify key barriers of timely initiation of complementary feeding.

## Abbreviations

AOR: Adjusted Odds Ratio
COR: Crude Odds Ratio
WHO: World Health Organization
ANC: Antenatal care
CI: Confidence interval
IYCF: Infant and young child feeding
DDS: Dietary Diversity Score
SD: Standard deviation
UNESCO: United Nations Educational, Scientific, and Cultural Organization

## References

[1] WHO (2010). Indicators for assessing infant and young child feeding practices PArt 3 Country Profiles.

[2] Khanal V, Sauer K, Zhao Y (2013). Determinants of complementary feeding practices among Nepalese children aged 6–23 months: findings from demographic and health survey 2011. BMC Pediatr.

[3] World Health Organization (2008). Indicators for assessing infant and young child feeding practices: part 1: definitions: conclusions of a consensus meeting held 6-8 November 2007 in Washington DC, USA.

[4] UNICEF, WHO (2007). Planning guide for national implementation of the Global Strategy for Infant and Young Child Feeding.

[5] WHO, UNICEF: Global strategy for infant and young child feeding. Geneva: World Health Organization; Geneva, Switzerland. 2003.

[6] Black R, Alderman H, Bhutta Z, Gillespie S, Haddad L, Horton S: Executive Summary of The Lancet Maternal and Child Nutrition Series. Maternal and Child Nutrition Study Group (eds), Maternal and Child Nutrition 2013:1-12. 10.1016/S0140-6736(13)60988-5 23746778

[7] UNICEF, WHO (2008). Strengthening action to improve feeding of infants and young children 6-23 months of age in nutrition and child health programmes: report of proceedings, Geneva.

[8] Khan ME, Donnay F, Tarigopula UK, Aruldas K (2013). Shaping demand and practices to improve family health outcomes: findings from a quantitative survey.

[9] Jones G, Steketee RW, Black RE, Bhutta ZA, Morris SS, Group BCSS (2003). How many child deaths can we prevent this year?. Lancet.

[10] WHO (2003). A tool for assessing national practices, policies and programmes Infant and Young Child Feeding.

[11] Saleh F, Ferdous Ara M, Hoque A, Alam MS (2014). Complementary feeding practices among mothers in selected slums of Dhaka city: a descriptive study. J Health Popul Nutr.

[12] Sinhababu A, Mukhopadhyay DK, Panja TK, Saren AB, Mandal NK, Biswas AB (2010). Infant-and young child-feeding practices in Bankura district, West Bengal, India. J Health Popul Nutr.

[13] Ogunlesi T, Ayeni V, Adekanmbi A, Fetuga B (2015). Determinants of timely initiation of complementary feeding among children aged 6‑24 months in Sagamu, Nigeria. Niger J Clin Pract.

[14] Save the children Ethiopia (2009). Ethiopia national nutrition strategy, review and analysis of progress and gaps: one year on.

[15] Ethiopia CSA, Macro I: Ethiopia demographic and health survey 2011. Addis Ababa, Ethiopia and Calverton, Maryland, USA: Central Statistical Agency (Ethiopia) and ICF International 2012.

[16] Semahegn A, Tesfaye G, Bogale A (2014). Complementary feeding practice of mothers and associated factors in Hiwot Fana Specialized Hospital, Eastern Ethiopia. Pan Afr Med J.

[17] Shumey A, Demissie M, Berhane Y (2013). Timely initiation of complementary feeding and associated factors among children aged 6 to 12 months in Northern Ethiopia: an institution-based cross-sectional study. BMC Public Health.

[18] Yemane S, Awoke T, Gebreslassie M (2014). Timely initiation of complementary feeding practice and associated factors among mothers of children aged from 6 to 24 months in Axum town, north Ethiopia. Int J Nutr Food Sci.

[19] Agedew E, Demissie M, Misker D, Haftu D: Early Initiation of Complementary Feeding and Associated Factors among 6 Months to 2 Years Young Children, in Kamba Woreda, South West Ethiopia: A Community–Based Cross-Sectional Study. J Nutr Food Sci. 2014;4(6):314. doi:10.4172/2155-9600.1000314.

[20] Kimani-Murage EW, Madise NJ, Fotso J-C, Kyobutungi C, Mutua MK, Gitau TM, Yatich N (2011). Patterns and determinants of breastfeeding and complementary feeding practices in urban informal settlements, Nairobi Kenya. BMC Public Health.

[21] Mihrshahi S, Kabir I, Roy S, Agho KE, Senarath U, Dibley MJ, Network SAIFR (2010). Determinants of infant and young child feeding practices in Bangladesh: secondary data analysis of Demographic and Health Survey 2004. Food Nutr Bull.

[22] Senarath U, Agho KE, Akram De S, Godakandage SS, Hazir T, Jayawickrama H, Joshi N, Kabir I, Khanam M, Patel A (2012). Comparisons of complementary feeding indicators and associated factors in children aged 6–23 months across five South Asian countries. Matern Child Nutr.

[23] Khanal V, Sauer K, Zhao Y (2013). Exclusive breastfeeding practices in relation to social and health determinants: a comparison of the 2006 and 2011 Nepal Demographic and Health Surveys. BMC Public Health.

[24] Federal Ministry of Health (2004). Ethiopian National Strategy on infant and young child feeding.

[25] Mekbib E, Shumey A, Ferede S, Haile F (2014). Magnitude and factors associated with appropriate complementary feeding among mothers having children 6–23 months-of-age in Northern Ethiopia; A Community-Based Cross-Sectional Study. J Food Nutr Sci.

[26] Rao S, Swathi P, Unnikrishnan B, Hegde A (2011). Study of complementary feeding practices among mothers of children aged six months to two years—a study from coastal south India. Australas Med J.

[27] Kabir I, Khanam M, Agho KE, Mihrshahi S, Dibley MJ, Roy SK (2012). Determinants of inappropriate complementary feeding practices in infant and young children in Bangladesh: secondary data analysis of Demographic Health Survey 2007. Matern Child Nutr.

[28] Seid AM, Yesuf ME, Koye DN (2013). Prevalence of Exclusive Breastfeeding Practices and associated factors among mothers in Bahir Dar city, Northwest Ethiopia: a community based cross-sectional study. Int Breastfeed J.

[29] Rez-Escamilla RP, Lãoetter C, Segall AM, Rivera A, Trevino-Siller S, Sanghvf T (1995). Exclusive breast-feeding duration is associated with attitudinal, socioeconomic and biocultural determinants in three Latin American Countries. J Nutr.

[30] Ogunba BO (2015). Effect of maternal employment on infant feeding practices in Southwestern Nigeria. Food Nutr Sci.

[31] Adnan N, Muniandy ND (2012). The relationship between mothers’ educational level and feeding practices among children in Selected Kindergartens in Selangor, Malaysia: A Cross-sectional Study. Asian J Clin Nutr.

[32] Tromp II, Briede S, Kiefte-de Jong J, Renders C, Jaddoe V, Franco O, Hofman A, Raat H, Moll H (2013). Factors associated with the timing of introduction of complementary feeding: the Generation R Study. Eur J Clin Nutr.

[33] Liaqat P, Rizvi MA, Qayyum A, Ahmed H, Ishtiaq N (2006). Maternal education and complementary feeding. Pak J Nutr.

[34] Abuya BA, Ciera J, Kimani-Murage E (2012). Effect of mother’s education on child’s nutritional status in the slums of Nairobi. BMC Pediatr.

[35] Wu Q, Scherpbier RW, van Velthoven MH, Chen L, Wang W, Li Y, Zhang Y, Car J (2014). Poor infant and young child feeding practices and sources of caregivers’ feeding knowledge in rural Hebei Province, China: findings from a cross-sectional survey. BMJ open.

[36] Manikyamba D, Vidya D, Satyavani A, Prasad AK, Deepthi KT (2015). Impact of nutritional education on the knowledge of mothers regarding infant and young child feeding practices. Scholars J Appl Med Sci.

[37] Hackett KM, Mukta US, Jalal CS, Sellen DW (2015). A qualitative study exploring perceived barriers to infant feeding and caregiving among adolescent girls and young women in rural Bangladesh. BMC Public Health.

[38] Kushwaha KP, Sankar J, Sankar MJ, Gupta A, Dadhich J, Gupta Y, Bhatt GC, Ansari DA, Sharma B (2014). Effect of peer counselling by mother support groups on infant and young child feeding practices: the Lalitpur experience. PLoS One.

[39] Bahemuka GJ, Munyanshongore C, Birungi F (2013). Knowledge, attitudes and practices of exclusive breast-feeding of infants aged 0-6 months by Urban refugee women in Kigali.

[40] Biks GA, Tariku A, Tessema GA (2015). Effects of antenatal care and institutional delivery on exclusive breastfeeding practice in northwest Ethiopia: a nested case–control study. Int Breastfeed J.

[41] Aidam BA, Perez-Escamilla R, Lartey A, Aidam J (2005). Factors associated with exclusive breastfeeding in Accra, Ghana. Eur J Clin Nutr.

[42] Bekele Y, Mengistie B, Mesfine F (2014). Prelacteal feeding practice and associated factors among mothers attending immunization clinic in Harari Region public health facilities, Eastern Ethiopia. Open J Prev Med.

[43] Legesse M, Demena M, Mesfin F, Haile D (2014). Prelacteal feeding practices and associated factors among mothers of children aged less than 24 months in Raya Kobo district, North Eastern Ethiopia: a cross-sectional study. Int Breastfeed J.

[44] Legesse M, Demena M, Mesfin F, Haile D (2015). Factors associated with colostrum avoidance among mothers of children aged less than 24 months in Raya Kobo district, North-eastern Ethiopia: community-based cross-sectional study. J Trop Pediatr.

